# Comparing Deaths from Influenza H1N1 and Seasonal Influenza A: Main Sociodemographic and Clinical Differences between the Most Prevalent 2009 Viruses

**DOI:** 10.1155/2012/501784

**Published:** 2012-12-30

**Authors:** German Fajardo-Dolci, Juan Pablo Gutierrez, Heberto Arboleya-Casanova, Sebastian Garcia-Saiso

**Affiliations:** ^1^Ministry of Health, 06600 Mexico City, DF, Mexico; ^2^Center for Evaluation Research and Surveys, National Institute of Public Health, 62100 Cuernavaca, MOR, Mexico; ^3^Health Policy Unit, London School of Hygiene and Tropical Medicine, London WC1E 7HT, UK; ^4^Ixtapaluca Highly Specialized Regional Hospital, Ministry of Health, 56530 Ixtapaluca, MEX, Mexico

## Abstract

*Background*. During the 2009 spring epidemic outbreak in Mexico, an important research and policy question faced was related to the differences in clinical profile and population characteristics of those affected by the new H1N1 virus compared with the seasonal virus. *Methods and Findings*. Data from clinical files from all influenza A deaths in Mexico between April 10 and July 13, 2009 were analyzed to describe differences in clinical and socioeconomic profile between H1N1 and non-H1N1 cases. A total of 324 influenza A mortality cases were studied of which 239 presented rt-PCR confirmation for H1N1 virus and 85 for seasonal influenza A. From the differences of means and multivariate logistic regression, it was found that H1N1 deaths occurred in younger and less educated people, and among those who engage in activities where there is increased contact with other unknown persons (OR 4.52, 95% CI 1.56–13.14). Clinical symptoms were similar except for dyspnea, headache, and chest pain that were less frequently found among H1N1 cases. *Conclusions*. Findings suggest that age, education, and occupation are factors that may be useful to identify risk for H1N1 among influenza cases, and also that patients with early dyspnea, headache, and chest pain are more likely to be non-H1N1 cases.

## 1. Introduction 

Influenza virus is the cause of one of the most common infections worldwide. Its effect on the global population carries important economic, healthcare system, and human suffering consequences [1]. Influenza virus type A is highly contagious and is the most pathogenic of all human influenza viruses [1]. 

Human antibodies recognize two antigens (glycoprotein) expressed on the viral surface called hemagglutinin (HA) and neuraminidase (NA). Changes in these glycoprotein represent antigenic variation (antigen drift) that is responsible for the constant changes within the common strains of seasonal influenza. Influenza is classified into 16 HA subtypes and 9 NA subtypes [1], and as pointed out, influenza comprises “the oldest emerging virus that is still emerging” [2]. 

Influenza pandemics occur when an influenza virus, that presents an hemagglutinin (HA) molecule for which there is limited or no existing immunity, emerges and efficiently transmits from human to human [3]. The emergence of a new virus subtype with a new HA is called antigen shift and will condition the lack of immune response to infection by the new virus.

Despite of pandemics during the last century, this disease generally does not represent an actual concern for the overall population. Several publications [1, 2, 4–7], as well as the World Health Organization (WHO) and the American Center of Disease Control (CDC), had repeatedly pointed out that in a manner of time, the world would face a new influenza pandemic that would cause significant figures of global morbidity and mortality.

Influenza has a fast person-to-person transmission and a high mortality risk when not treated. Among all the major pandemics, influenza A is the only one that may potentially infect a considerable fraction of the world's population in a few months [2].

The genomes of the last three pandemic influenza viruses, 1918 (H1N1), 1957 (H2N2), and 1968 (H3N2), originated from nonhuman reservoirs and all HA genes originated from avian influenza viruses [3].

There are two mechanisms intervening in the introduction of a virus with new hemagglutinin (HA) subtypes in the human population: recombination and interspecies transmission. The first mechanism of the current Influenza A (H1N1) pandemic comprises recombined viruses of two swine flu types: one of those has a triple recombination strain containing segments originated from the last human seasonal flu H3N2 and the other one originated by avian and swine flu [4]. 

In early April 2009, cases similar to pneumonia and influenza were notified and reported to the Pan-American Health Organization (PAHO). As research progressed, it was observed that reported Mexican and Californian cases were caused by a similar virus, which triggered the alert of the World Health Organization (WHO) on April 24. On July 11, 2009, the WHO raised its pandemic alert level to 6. 

At the beginning of the outbreak there were a significant number of cases reported as possible new H1N1 influenza that, after the PCR test became widely available in May 2009, were labeled as seasonal influenza A (non-H1N1). The National Commission for Medical Arbitration (CONAMED), as the designated authority to monitor all mortality cases due to influenza, compiled a series of clinical files of all suspicious cases. Since the clinical profile of the new H1N1 virus is not significantly different to that of seasonal influenza A (non-H1N1)—described clinical profiles include cough, rhinorrhea, headache, myalgia, arthralgia, fever, dyspnea, and diarrhea [8–13]—many of the initial cases filed by CONAMED were later confirmed by rt-PCR testing as seasonal influenza A (non-H1N1).

This study describes the main differences between the new influenza H1N1 and seasonal influenza A virus mortality cases in terms of clinical profile and sociodemographic characteristics which may orient clinicians and authorities in decision-making processes.

## 2. Methodology

This is a cross-sectional analysis of secondary data related to deaths due to influenza A infection during the period from April 10 to July 13, 2009. Cases of H1N1 and seasonal influenza A (non H1N1) were identified, as well as information on the clinical and socio-demographic characteristics of individuals. For this study, “case” was considered as any deaths due to influenza type A confirmed by the National Institute of Epidemiologic Diagnosis and Reference (INDRE) through the rt-PCR test for influenza A (H1N1) or seasonal influenza A. The analysis was based on information collected from patients' clinical records and reporting forms from health facilities in Mexico.

As a first step to analyze the deaths from influenza A H1N1, the necessary coordination with health care institutions and States of Mexico was established in order for them to send records of all patients who had died with clinical diagnosis of influenza across the country to CONAMED [14]. The influenza working group decided to develop two methods of analysis for such records, a clinical and an epidemiological one. The first in order to identify the individual characteristics of the deceased as well as the process of medical care received, the second to know the general characteristics of the population affected by variables of time, place, and person, and to estimate the rate of mortality and early lethality. In both cases, the primary source document was the clinical file of deceased patients diagnosed with influenza A (H1N1 and non-H1N1) during the period from April 10 to July 13, 2009.

Once a database including only information from influenza A deceased patients with confirmation of whether it was H1N1 or seasonal A was constructed, variables needed to describe socio-demographic and clinical characteristics were reviewed to check consistency. 

The differences in the socio-demographic profile of the population were analyzed, seeking to identify characteristics that might be associated with a greater likelihood of death from H1N1 compared to influenza type A, that is, trying to identify a profile for these patients. For this purpose, tests for differences of means or proportions were calculated between groups, and further multivariate logistic regression models were carried out.

To identify possible clinical differences among reported deaths and in order to avoid bias due to differences in socio-demographic characteristics, cases of H1N1 were matched on these socio-demographic characteristics to cases of non-H1N1 using propensity score matching techniques. The propensity score was constructed using age, sex, economic activity, schooling, and marital status. Smoking was included as it was assumed it could be related to the outcomes.

Estimations were implemented using kernel matching in order to utilize as much information as possible from the available observations. The kernel matching compared a case (H1N1) to the average of the noncases (non H1N1) that had a score in the proximity of the case; in this way, for all cases a match was generated.

Analysis was implemented with Stata 10. This study was approved by the National Commission for Medical Arbitration Ethics Committee.

## 3. Results

The sample included 324 influenza A cases of which 239 had rt-PCR diagnosis of influenza A H1N1 and 85 of seasonal influenza A (non H1N1). Of the total 53.1% were women and 46.9% men. Within the influenza H1N1 group 50.6% were women and 49.4% men. Within the seasonal influenza A group, 60% were women and 40% men. 41% of deaths within the influenza A H1N1 group occurred in the 20–39-years-old age group and the same age group represented also 41% of the seasonal influenza A group.

In terms of the general characteristics of individuals within the entire sample, the analysis shows that there are differences by age, education, and type of occupation among those who presented AH1N1 influenza in relation to influenza A non H1N1. Comparing between the two types of influenza, H1N1 deaths would occur from an earlier age, in people with less education, and among those who engage in activities where there is increased contact with other unknown persons (trade or other independent activities) (Table 1).

Clinical manifestations and outcomes were similar in both types of influenza in most of the indicators but dysnea, headache, and chest pain that were less frequently found among cases with H1N1 compared to non H1N1 (Table 2).

For the regression analysis, the differences in some socio-demographic indicators were maintained in the multi-variable models. As reported in Table 3, junior high school was associated with lower probability of H1N1 compared to primary school or less and also, having an economic activity related to increased contact with unknown individuals (salespersons or independent professionals) was associated with an increased probability of H1N1 (OR 4.52 95% CI 1.56–13.14) (Table 3).

To analyze the occurrence of clinical features in deaths, controlling for socio-demographic characteristics of individuals, as described in the methods section, a matched analysis was implemented. After matching, cough was more prevalent among those with H1N1 influenza, while dyspnea, headache, and chest pain were less prevalent among H1N1 compared wit non H1N1. Also, it was found that days from clinical care to death were in average more for those with H1N1. In all other clinical features analyzed, including time elapsed between diagnosis and treatment, and between diagnosis and death, there were no significant differences (Table 4).

## 4. Discussion

The results presented here describe some differences among deceased individuals with influenza H1N1 compared to those with seasonal influenza A (non H1N1). Cases of H1N1 were younger and in average less educated, confirming observations made during the peak of the pandemic [15]. A contextual factor that could be related to higher exposure is the primary economic activity: cases of H1N1 were individuals that reported in a larger proportion working as salespersons or activities with a higher interaction with unknown individuals, that could result in a larger variety of exposure.

In a previous study that analyzed the first 120 mortality cases of the new H1N1 virus [16], it was found that a proportion of 51% were women and 49% were males. 45.1% of cases occurred in patients aged between 20 and 39. The mortality rate was 2.2% and ranged from 0.3% in the 10 to 19 years and 6.3% for 50 to 59 years old group, which coincides with our initial findings. Up to this point the question of which were the main differences between the new influenza H1N1 virus infection and the infection by seasonal A virus was still unanswered.

Most published case series have shown similarities between infection by both H1N1 and seasonal influenza A, main symptoms being fever, cough, headache, myalgia, and rhinorrhea [8–10, 12–14, 17, 18]. In terms of clinical characteristics that differentiate between the two groups, it was found that cough was more prevalent among those with H1N1 influenza, while dyspnea, headache, and chest pain were less prevalent among H1N1 compared to seasonal A influenza virus. This particular finding is worth noting since these are symptoms which may encourage patients to seek medical care and help physicians diagnose and determine severity of the case as it has been described as signs of progression to more severe disease or complications [19]. While coughing was more prevalent, which is unspecific for most uncomplicated upper airway infections, symptoms that would alert of a complicated case were not as prevalent, this could be the reason behind the perceived lack of opportune treatment reported by many during the start of the pandemic in June–August 2009 [14]. 

Also, it was found that days from clinical care to death were in average more for those with infection by H1N1 virus, signaling a relative delay in the evolution of the clinical profile after receiving medical care, which may be related to the H1N1 susceptibility and therefore response to antiviral therapy, although in severe cases only a partial one. It also correlates to longer evolution times and presence of virus for longer periods reported by others [19].

The present study shows the main differences in the mortality cases between the new influenza H1N1 and seasonal influenza A virus in terms of clinical profile and socio-demographic characteristics on mortality cases; these differences may prove useful to orient clinicians and authorities in decision making processes.

At the present time, morbidity and mortality rates from the H1N1 virus have dropped; however, the need to investigate the characteristics of this novel influenza virus along with the epidemiological pattern of the pandemic is still patent. This will increase our understanding of its nature and may help determine future plans to be prepared in case of similar events. As pointed out by the WHO, most studies drawing comparisons between influenza A H1N1 and seasonal influenza A mortality are based on estimates and related to differences in mortality rates [20], comparisons that may prove to be inaccurate and sometimes misleading. The present study does not pretend to conclude on these, but to draw attention to the main sociodemographic and clinical differences of both influenza A infections. 

## Figures and Tables

**Table 1:** Sociodemographic characteristics of deceased cases, by type of influenza.

Variable	Non-H1N1	H1N1	*P* value *t*-test
Age (means)	38.26	33.07	**0.038**
Sex (men)	60%	51%	0.135
Smoking	21%	15%	0.259
Have a partner	60%	49%	0.090
Primary school or less	54%	60%	0.331
Junior high school	26%	13%	**0.018**
High school or higher level	20%	26%	0.224
Housewife	33%	23%	0.079
Unemployed or retiree	14%	8%	0.134
Student	11%	20%	**0.036**
Independent worker, salesperson	8%	25%	**0.000**
Employee	33%	24%	0.124

**Table 2:** Clinical characteristics of deceased cases, by type of influenza.

Variable	Non-H1N1	H1N1	*P* value *t*-test
Pneumonia	33%	43%	0.125
Fever	84%	85%	0.826
Cough	81%	87%	0.229
Dyspnea	88%	73%	**0.001**
Expectoration	50%	48%	0.721
Myalgia	38%	31%	0.239
Rhinorrhea	28%	26%	0.769
Cyanosis	26%	25%	0.869
Headache	52%	29%	**0.000**
Chest pain	37%	15%	**0.000**
Nasal obstruction	5%	9%	0.196
Vomiting	6%	10%	0.189
Diarrhea	9%	9%	0.945
Days from symptoms start to care	6.86	6.30	0.582
Days from care to death	6.86	7.79	0.304
Days from symptoms start to death	13.96	14.03	0.962

**Table 3:** Regression analysis.

Variable	OR (95% CI)
Sex (women)	1
Sex (men)	0.762 (0.389–1.494)
Age	0.989 (0.971–1.007)
Have a partner	0.789 (0.430–1.448)
Smoking	0.722 (0.350–1.489)
Primary school or less	1
Junior high school	**0.391** **(0.189–0.809)**
High school or higher level	1.651 (0.766–3.559)
Housewife	1
Unemployed or retiree	0.674 (0.242–1.878)
Student	1.165 (0.359–3.778)
Independent worker, salesperson	**4.523** **(1.556–13.144)**
Employee	0.664 (0.280–1.574)

**Table 4:** Average differences after matching (H1N1 as exposure).

Variable	Matched difference	*t*-stat
Pneumonia	0.0804	1.02
Fever	−0.0303	−0.54
Cough	0.1138	**1.78**
Dyspnea	−0.1318	−**2.30**
Expectoration	−0.0469	−0.58
Myalgia	−0.0704	−0.91
Rhinorrhea	−0.0560	−0.79
Cyanosis	−0.0117	−0.16
Headache	−0.0210	−**2.51**
Chest pain	−0.1591	−**2.09**
Nasal obstruction	0.0512	1.46
Vomiting	0.0212	0.50
Diarrhea	0.0083	0.17
Days from symptoms start to care	0.2744	0.20
Days from care to death	1.8296	**1.63**
Days from symptoms start to death	1.8925	1.05
